# The difficult doctor? Characteristics of physicians who report frustration with patients: an analysis of survey data

**DOI:** 10.1186/1472-6963-6-128

**Published:** 2006-10-06

**Authors:** Erin E Krebs, Joanne M Garrett, Thomas R Konrad

**Affiliations:** 1 Center for Implementing Evidence-Based Practice, Richard L. Roudebush Veterans Affairs Medical Center, Indianapolis, IN, USA; 2 Department of Medicine, Indiana University, Indianapolis, IN, USA; 3 Department of Obstetrics and Gynecology, University of North Carolina at Chapel Hill, Chapel Hill, NC, USA; 4 Cecil G. Sheps Center for Health Services Research, University of North Carolina at Chapel Hill, Chapel Hill, NC, USA

## Abstract

**Background:**

Literature on difficult doctor-patient relationships has focused on the "difficult patient." Our objective was to determine physician and practice characteristics associated with greater physician-reported frustration with patients.

**Methods:**

We conducted a secondary analysis of the Physicians Worklife Survey, which surveyed a random national sample of physicians. Participants were 1391 family medicine, general internal medicine, and medicine subspecialty physicians. The survey assessed physician and practice characteristics, including stress, depression and anxiety symptoms, practice setting, work hours, case-mix, and control over administrative and clinical practice. Physicians estimated the percentage of their patients who were "generally frustrating to deal with." We categorized physicians by quartile of reported frustrating patients and compared characteristics of physicians in the top quartile to those in the other three quartiles. We used logistic regression to model physician characteristics associated with greater frustration.

**Results:**

In unadjusted analyses, physicians who reported high frustration with patients were younger (p < 0.001); worked more hours per week (p = 0.041); and had more symptoms of depression, stress, and anxiety (p < 0.004 for all). In the final model, factors independently associated with high frustration included age < 40 years, work hours > 55 per week, higher stress, practice in a medicine subspeciality, and greater number of patients with psychosocial problems or substance abuse.

**Conclusion:**

Personal and practice characteristics of physicians who report high frustration with patients differ from those of other physicians. Understanding factors contributing to physician frustration with patients may allow us to improve the quality of patient-physician relationships.

## Background

Approximately 15% of patient encounters in adult primary care settings are unusually difficult from the physician's perspective[1,2]. Most of the literature related to these difficult doctor-patient encounters has focused on the patient, rather than on the physician or practice setting involved. Patients who are perceived as difficult or frustrating are more likely than other patients to have psychiatric conditions, abrasive personality traits, and personality disorders [1-5]. They are more likely to report multiple physical symptoms, which are often medically unexplained[1,2,6]. Not surprisingly, these patients are also more frequently dissatisfied with their medical care[2,7].

Although it is generally acknowledged that physicians share responsibility for difficult relationships, characteristics of physicians who report frustration with patients are not well defined. Data about the influence of practice settings and health care systems on perceived difficulty of patient-physician encounters are also scarce. It has been suggested that system factors such as time pressure, administrative burdens, and lack of control over clinical care may lead to more physician frustration with patients, especially in managed care settings [8-10]. The objective of this study was to describe physician and practice characteristics associated with greater physician-reported frustration with patients.

## Methods

The Physician Worklife Survey measured personal and practice characteristics and work satisfaction of a national sample of US physicians in 1996–97. The survey was developed in a multi-step process and pilot tested with a large sample of physicians. Detailed descriptions of this process have been published [11-13]. The Physician Worklife Survey was mailed to a national stratified random sample of physicians in family medicine, internal medicine and pediatric specialties, selected from the American Medical Association Masterfile. The sample was stratified by race, specialty, and regional level of participation in managed care. Physicians returned 2326 usable surveys, yielding an adjusted response rate of 52% after correction for incorrect addresses, refusals, and ineligible responses[11]. We excluded pediatricians and pediatric subspecialists from our analysis, leaving 1391 eligible family medicine, general internal medicine, and medical subspecialist physicians.

### Measurements

#### Frustration with patients

Physicians were asked to estimate the percentage of patients in their practices who were "generally frustrating to deal with." We categorized their responses into quartiles and defined physicians in the top quartile as "highly frustrated."

#### Physician and practice variables

Participants rated their general health on a 5-point scale from poor to excellent. They reported how often, on a 5-point scale, they felt "sad or depressed" in the past year. We refer to physicians as "often depressed" if they reported symptoms occurring fairly or very often. Participants also reported how often they felt "anxious or nervous." We referred to them as "often anxious" if they reported symptoms fairly or very often. Stress was measured using the 4-item version of the Perceived Stress Scale[15]. We calculated the total number of hours worked per week by summing the hours spent seeing patients in the clinic and in the hospital, performing other patient-related activities (such as paperwork and phone calls), and doing other work-related activities (such as administration or teaching).

#### Physician control scales

A total of 12 items measuring perceived control over aspects of practice were included in the survey. We performed principal component analysis with oblimin rotation using our sample of non-pediatricians (see Additional file 1). Ten items loaded on 2 factors, which we described as clinical control (4 items) and administrative control (6 items). The alpha coefficients for these scales were 0.702 and 0.803, respectively. The remaining 2 items, which could represent either administrative or clinical domains depending on the work context, had cross loadings on both factors and were excluded from the control scales. We imputed missing values of component items using best subset regression when only 1 value from a scale was missing, and excluded responses if >1 value was missing.

#### Patient case-mix variables

Physicians were asked to report the percentage of patients in their practices who were white, black, Hispanic, Native American/Alaskan, and Asian/Pacific Islander. For our analysis, we used a dichotomous variable of percent white/non-white patients. Physicians reported the percentage of their patients who were uninsured, who received Medicaid, who had substance abuse problems, and who had "complex or numerous" medical problems or psychosocial problems.

### Data analysis

We used Pearson's chi-square tests to compare characteristics of "highly frustrated" physicians to those of other physicians. We fit multivariable logistic regression models using a two-stage strategy to assess characteristics associated with greater physician-reported frustration with patients. In the first stage, we modeled associations between frustration and physician and practice characteristics, which included all physician and practice variables from the bivariable analyses. We used multiple degree-of-freedom Wald tests to remove groups of variables that were not significantly associated with high frustration. Secondly, we added patient case-mix variables (percent of patients who are white, who are uninsured, who receive Medicaid, who have substance abuse problems, who have complex medical problems, and who have complex psychosocial problems) that might explain any of the observed associations between physician and practice characteristics and frustration. Case-mix variables that did not confound these relationships were removed from the final model. The final model included the set of variables that were statistically significant (p < 0.05) after adjustment for other variables in the model. Results did not substantially differ when continuous variables in the final model were categorized into dichotomous variables. Therefore, we used the dichotomous form of the variables for ease of interpretation, reporting percentages for the bivariate analyses and odds ratios (OR) and 95% confidence intervals (CI) for the logistic regression models. We adjusted all analyses for sampling weights and strata included in the sample design using the statistical package Stata version 8.0 (Stata Corp, College Station, TX).

## Results

**Figure 1:**
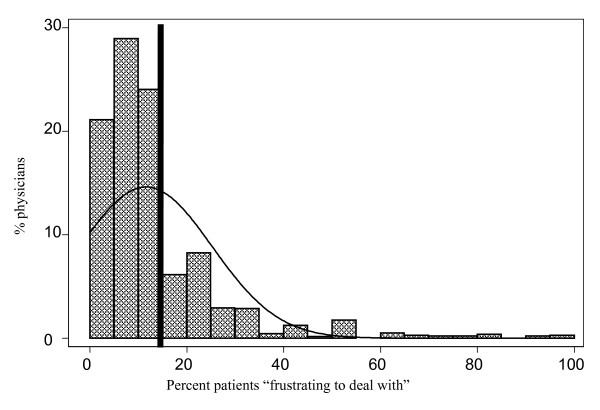
**Distribution of reported percent of patients who are frustrating**. The bold line marks the 75^th ^percentile; we defined physicians to the right of the lines as "highly frustrated."

Table 1 shows personal and practice characteristics of the 1391 physician participants. Their mean age was 47 years, and most were male (77%) and white (82%). Forty percent worked in a small group practice setting, 21% worked in solo practice, 18% in a large group practice, 7% in academic medicine, and 6% in a group or staff model health maintenance organization. 12% of physicians were often anxious, 10% were often depressed. Physicians worked a mean of 55 hours per week. Physicians reported, on average, that 12% of their patients were "generally frustrating to deal with." Those who reported that >15% of their patients were frustrating were in the top quartile (Figure 1).

In the unadjusted analysis, all of the personal physician characteristics, with the exception of physician race (p = 0.96), were significantly related to high frustration (Table 2). Similarly, nearly all of the practice characteristics assessed were significantly associated with high frustration (Table 3). Physicians who practiced in group or staff model health maintenance organizations (HMO) and those who practiced in miscellaneous settings (such as urgent care or emergency departments) were more likely than physicians in other settings to be highly frustrated. Having less reported control over administrative aspects of practice was associated with greater frustration (p < 0.001). The association between control over clinical practice and frustration was of borderline statistical significance (p = 0.053).

### Multivariable model results

#### Physician and practice characteristics

In the model considering physician and practice characteristics alone (without case-mix), the characteristics independently associated with high frustration were younger age, higher stress, greater number of hours worked per week, medicine subspecialty, HMO practice setting, and miscellaneous practice setting (such as urgent care).

#### Patient case-mix

When we added case-mix variables to the model, the only variables with an effect were percentage of patients with substance abuse (p = 0.024) and percentage of patients with complex psychosocial problems (p < 0.001).

#### Final model

The final multivariable model of characteristics independently associated with high frustration included younger age, higher stress, more hours worked per week (borderline significance), medicine subspecialty, and higher reported percentages of patients with psychosocial and substance abuse problems (Table 4). Practice setting was no longer significantly associated with high frustration when adjusted for case-mix.

Younger physicians and those with above-average stress had greater odds of frustration. Specialty was also independently associated with frustration; both subspecialists and general internists appeared to have greater odds of high frustration than family physicians, but this difference only reached statistical significance for medicine subspecialists (OR = 2.0, 95% CI 1.3–3.3). After adjustment for case-mix, the association between longer work hours and frustration was of borderline statistical significance (OR = 1.5, 95% CI 1.0–2.2).

### Additional analysis

Moderate correlation was present between the anxiety, depression, and stress variables (r = 0.36–0.58). To assess for independent associations between these variables and high frustration, we evaluated 3 additional logistic regression models, excluding all but 1 of the 3 correlated variables at a time (data not shown). Each of the individual variables was significantly associated with frustration when the other 2 variables were excluded; this did not substantially change results for other variables in the full model.

We also conducted analyses evaluating physicians in the top decile of reported frustrating patients (those who reported more than 25% of their patients were frustrating to deal with), but found similar results to those we have reported using our quartile definition.

## Discussion

Although the difficult doctor-patient relationship has been a focus of inquiry for many years, research has largely focused on characteristics of "difficult patients." Personal and practice characteristics of physicians who report frustration with patients have received less attention. We found that physicians vary substantially in the percentage of patients they perceive to be frustrating, and that physicians who report a high percentage of frustrating patients differ from other physicians in a number of ways. In particular, highly frustrated physicians were younger, more likely to practice subspecialty internal medicine, and more likely to have high stress. Some of the variability we observed between physicians may be due their differing tendencies to perceive a given patient as frustrating. However, physician behaviors may also affect reported frustration with patients. The clinical encounter is a dynamic process, and physician behaviors, especially communication methods, may alter the character of a patient encounter in ways that promote or alleviate interpersonal difficulty.

We found that physicians who reported more patients with psychosocial problems and with substance abuse problems were more likely to be highly frustrated, while those who reported more patients with complex medical problems were not. This finding is consistent with studies of difficult doctor-patient encounters, which have consistently found that patients with psychiatric illness are more likely to be perceived as difficult by their physicians [1-5]. Physicians in general are better prepared by training to address biomedical problems than psychosocial problems[17,18]. However, individual physicians vary in their perspectives on the importance of psychosocial care. This variation may explain some of the differences we observed between physicians. In one study involving 38 primary care physicians practicing in a walk-in clinic, investigators found that physicians with negative beliefs about the value of psychosocial care rated more encounters as difficult[2]. Notably, patients involved in these difficult encounters had worse outcomes in terms of less satisfaction with the visit, more unmet expectations for care, and higher subsequent health care utilization.

Physician beliefs about psychosocial aspects of practice may be a factor in the association we observed between specialty and frustration. We found that family physicians were less likely to report high frustration with patients than physicians in both general internal medicine and medicine subspecialties, although this association reached statistical significance only for subspecialists. The philosophy of family medicine embraces a relatively holistic approach to patient care, [19] and there is some evidence that family physicians may have more favorable beliefs about psychosocial care than internists [20].

Older physicians were less likely to report high frustration with patients, perhaps because of greater clinical experience or a more flexible and humanistic approach to patient care. It is also possible that there are fewer highly frustrated physicians in the older age group because these physicians are less likely to continue practicing clinical medicine.

In our preliminary exploration of the relationship between practice factors and frustration with patients, we evaluated practice factors that have been hypothesized to be potential contributors to difficult doctor-patient relationships, including length of appointment time, type of practice, number of hours worked per week, and perceived control over administrative and clinical issues. The association between frustration and HMO or miscellaneous practice setting was no longer statistically significant after adjustment for case-mix. Only number of hours worked per week was associated with frustration in the full model; this association had borderline statistical significance after adjustment for case-mix. The effect of prolonged work hours on physician wellbeing, physician-patient relationships, and patient safety is an area of ongoing interest, especially in the context of policies limiting resident physician work hours. We speculate that working long hours may decrease tolerance for dealing with challenging patient issues, but this hypothesis requires further study.

Several limitations of our study deserve consideration. First, our data are based on physician report, so we are unable to determine how much of the variation in reported frustrating patients is due to differences between physicians' case-loads and how much is due to differences between physicians themselves. While some physicians certainly have more challenging patient panels, prior research has found that physicians report substantial differences in perceived difficulty with patients even when patients are new and arbitrarily assigned[2]. Second, our measures of physician mental health characteristics relied on brief measures. Detailed assessments of physician personality, mental health, and interpersonal skills were beyond the scope of this survey. Finally, because the data are cross-sectional, we cannot draw conclusions about the direction of relationships between physician factors and reported frustration with patients.

## Conclusion

Are the highly frustrated physicians we identified truly "difficult doctors"? More likely, they are a diverse group of doctors facing varied personal and clinical challenges. Among them may be physicians who are inadequately equipped to address the complex psychosocial needs of their patients. Ultimately, we need better understanding of the role that physicians play in frustrating doctor-patient relationships so we can develop strategies to improve the wellbeing of both patients and their doctors.

## Competing interests

The author(s) declare that they have no competing interests.

## Authors' contributions

EEK developed the study design, performed statistical analyses, and drafted the manuscript. JMG participated in the study design, performed statistical analysis, and edited the manuscript. TRK participated in the design and administration of the original survey, participated in the design of this secondary study, performed statistical analysis, and edited the manuscript. All authors read and approved the final manuscript.

## Pre-publication history

The pre-publication history for this paper can be accessed here:



## Supplementary Material

Additional file 1 Factor analysis of control measures. The table shows factor analysis results for survey items measuring physicians' perceived control over aspects of practice. Click here for file

## Figures and Tables

**Table 1:** Characteristics of participating physicians

**Physician characteristics**	Mean*	Percent*
Age, mean years	47	
Male		77
Race/Ethnicity		
White		82
Asian		10
Hispanic		4
Black		2
Other		2
Specialty		
Family medicine		47
General internal medicine		40
Medicine subspecialty		12
Fair-poor health		6
Often depressed		10
Often anxious		12

**Practice characteristics**		

Practice setting		
Small group		40
Solo		21
Large group		18
Miscellaneous		9
Academic		7
Health maintenance organization		6
Work-hours, mean hours/week	55	
Follow-up visit time, mean minutes	15	
Patients, estimated mean %		
with complex medical problems	42	
with psychosocial problems	29	
with substance abuse	9	

*Adjusted for survey weights and stratification. N = 1391.

**Table 2:** Personal characteristics of highly frustrated physicians

**Physician characteristic**	**Percent highly frustrated**	P value*
Age		
<40 years	40	<0.001
≥40 years	22	
Sex		
Male	24	0.04
Female	32	
Race		
White	26	0.96
Nonwhite	26	
Specialty		
Family medicine	21	0.01
General internal medicine	29	
Medicine subspecialty	34	
General health		
Good-excellent	25	0.02
Poor-fair	43	
Often depressed		
No	24	0.004
Yes	42	
Often anxious		
No	23	<0.001
Yes	47	
Stress		
Below average	16	<0.001
Above average	36	

* Pearson's chi-square, adjusted for sampling weights and stratification

**Table 3:** Practice characteristics of highly frustrated physicians

**Practice characteristic**	**Percent highly frustrated**	P value*
Practice setting		
Small group	22	0.01
Solo	22	
Large group	26	
Miscellaneous	38	
Academic	30	
Health maintenance organization	45	
Hours worked per week		
≤55 hours	22	0.02
>55 hours	31	
Time for routine visit		
<15 minutes	27	0.70
15+ minutes	25	
Pts w/complex med problems		
Fewer than average	19	<0.001
More than average	36	
Pts w/psychosocial problems		
Fewer than average	15	<0.001
More than average	40	
Pts w/substance abuse		
Fewer than average	15	<0.001
More than average	42	
Administrative control		
Below average	36	<0.001
Above average	17	
Clinical control		
Below average	29	0.053
Above average	23	

* Pearson's chi-square, adjusted for sampling weights and stratification

**Table 4:** Physician and practice characteristics associated with high frustration, multivariable model

**Characteristic**	Odds Ratio (95% CI)*
Age	
< 40 years	1.7 (1.1–2.7)
40 or more years	1.0
Stress	
Below average	1.0
Above average	2.3 (1.5–3.4)
Specialty	
Family medicine	1.0
General internal medicine	1.5 (0.9–2.3)
Medicine subspecialty	2.0 (1.3–3.3)
Hours worked per week	
≤55 hours	1.0
>55 hours	1.5 (1.0–2.2)
Patients with psychosocial problems	
Fewer than average	1.0
More than average	2.1 (1.4–3.2)
Patients with substance abuse	
Fewer than average	1.0
More than average	2.9 (1.8–4.5)

* Multivariable logistic regression, adjusted for weights and stratification (n = 1341).
