# Supplementary material for: The difficult doctor? Characteristics of physicians who report frustration with patients: an analysis of survey data
Source: BMC Health Serv Res. 2006 Oct 6;6:128. doi: 10.1186/1472-6963-6-128 (PMC1617099; doi:10.1186/1472-6963-6-128)
Supplement: Additional file 1 — Factor analysis of control measures. The table shows factor analysis results for survey items measuring physicians' perceived control over aspects of practice. [file 1472-6963-6-128-S1.doc]

**Additional file 1 — Factor analysis of control measures**

| **Items**  Control over… | | **Administrative Control** | **Clinical Control** |
| --- | --- | --- | --- |
|  | when to admit patients | 0.174 | **0.719** |
|  | length of hospital stay | 0.162 | **0.750** |
|  | choice of medications | 0.195 | **0.694** |
|  | choice of diagnostic tests | 0.184 | **0.731** |
|  | clinic schedule | **0.680** | 0.372 |
|  | volume of paperwork | **0.571** | 0.070 |
|  | hours of work | **0.751** | 0.166 |
|  | work interruptions | **0.762** | 0.114 |
|  | patient load | **0.818** | 0.210 |
|  | workplace issues | **0.663** | 0.220 |
| Alpha coefficient for control scales | | **0.803** | **0.702** |

Two ambiguous items were dropped from the scale because of cross loading on both types of control (“pre-authorization for necessary services,” “referral relationships”).
